# Bed Surge Capacity in Saudi Hospitals During the COVID-19 Pandemic

**DOI:** 10.1017/dmp.2021.117

**Published:** 2021-04-19

**Authors:** Fahad Alqahtani, Anas Khan, Jalal Alowais, Tareef Alaama, Hani Jokhdar

**Affiliations:** 1 General Directory for Emergency Management, Ministry of Health, Saudi Arabia; 2 Department of Emergency Medicine, King Saud University, Riyadh, Saudi Arabia; 3 Department of Surgery, Imam Muhammad ibn Saud Islamic University, Riyadh, Saudi Arabia; 4 Ministry of Health, Saudi Arabia

**Keywords:** general hospital bed, ICU bed, Saudi Arabia, surge capacity

## Abstract

**Objectives:**

To assess the hospital beds and intensive care unit (ICU) beds with a ventilator surge capacity of the health system in Kingdom of Saudi Arabia (KSA) during the coronavirus disease (COVID-19) pandemic.

**Methods:**

This study used relevant data from the National Health Emergency Operation Center to estimate general hospital and ICU bed surge capacity and tipping points under 3 distinct transmission scenarios.

**Results:**

The study results reveal that hospitals in the KSA need to be supplied with additional 4372 hospital beds to care for COVID-19 positive cases if the pandemic continues over a 6 months’ period. At the same time, it requires additional 2192 or 1461 hospital beds if the pandemic persists over a 12- or 18-month period, respectively, to manage hospitalized COVID-19 overloads. The health system surge capacity would suffer from a shortage of 1600, 797, and 540 ICU beds under the 3 transmission scenarios to absorb critical and intensive care COVID-19 cases.

**Conclusion:**

Our findings highlight the urgent need for additional hospital and ICU beds in the face of critical COVID-19 cases in KSA. The study recommends further assessment measures to the health system surge capacity to keep the Saudi health system prepared during the COVID-19 pandemic.

## Introduction

Pandemics generally exhibit a crucial challenge and a unique threat to the country’s health care system; they could overwhelm the health care system by the substantial increase in the demand and supply for health services.^1–3^ Therefore, pandemic preparedness necessitates proper infrastructure and capacity as a critical aspect of a country’s emergency response.^1^ The hospital bed surge capacity, which is usually defined as the capacity to accommodate and ingest the overwhelming demand for health care services, is one of the most critical issues during the health care system response to epidemics.^1,4,5^ Health care systems are generally prepared and designed to overcome the average demand for health services rather than acute demand caused by pandemics.^5^ In the recent flu seasons, it was reported that hospital emergency departments achieved their full capacity limits, with few rooms for a surge in patients in either emergency or inpatient beds.^6^ Health system surge capacity comprises 2 different broad components, including hard and soft components. The hard components consist of health workforces, such as nurses, doctors, emergency medical personnel, pharmacists, as well as infrastructure, equipment, and supplies. The soft components consist of a digital health system, such as well coordination and management, guidelines and protocols to prevent and mitigate infection rates, and effective communication.^7^

Coronavirus disease (COVID-19) is a severe respiratory viral infection that has quickly spread to most parts of the world, infecting millions of people and causing numerous mortalities.^8^ The COVID-19 pandemic has overwhelmed the health care services capacity in different parts of the world. The recent experience in various countries, such as China, Brazil, Italy, Iran, Spain, and the United States, has highlighted the significant need to ensure adequate inpatient and intensive care unit (ICU) beds capacity. Even though some of these countries, such as the United States and Spain, have well-resourced and robust health care services, they were unable to overcome the sudden surge in the relatively high number of confirmed COVID-19 cases requiring hospitalization and intensive care.^9,10^

**Figure 1.**
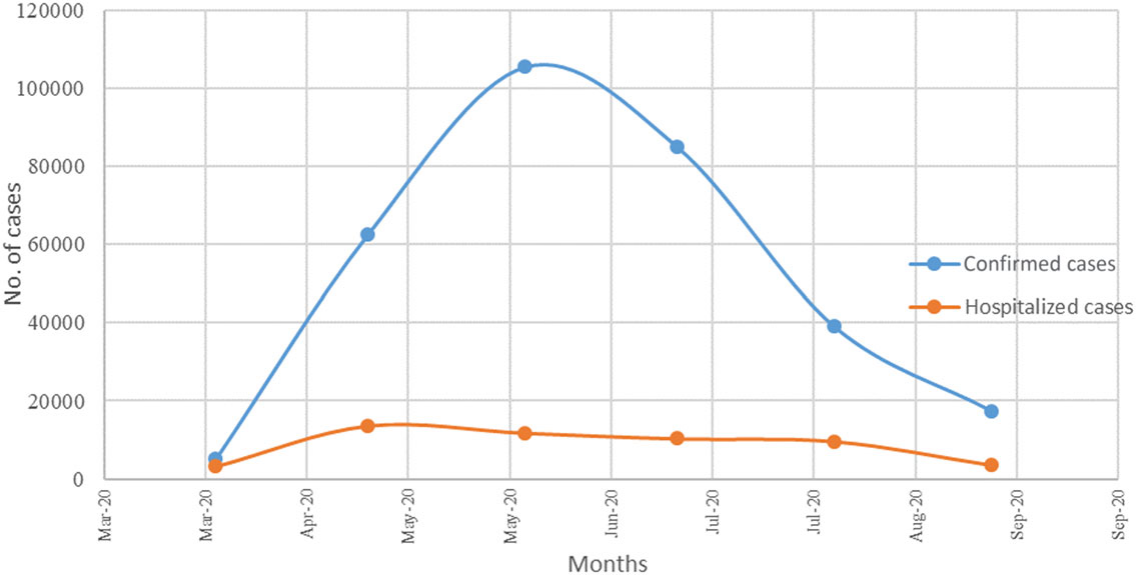
Number of confirmed cases versus number of hospitalized cases (April to September 2020).

The Kingdom of Saudi Arabia (KSA) is the largest country in the Arabian Peninsula. The total population size in KSA for the year 2020 is 34 810 000.^11^ Foreign residents account for more than one-third of the population size in KSA.^12^ As of March 2, 2020, the KSA has reported the first confirmed case of COVID-19; since then, the number of positive tested cases had increased gradually.^13^ KSA had 337 711 confirmed cases by October 7, 2020, of which 323 208 cases recovered at a rate of 95.7%, and 4947 deaths were reported at a rate of 1.5%. The rate of hospital admission of the COVID-19 patients ranged from 11% to 62% over the period from April to September 2020. The average hospitalization rate is 18% (CI: 5.6% to 45%; Figure 1; https://covid19.moh.gov.sa/). The Saudi authority has obliged mask-wearing in public places, alongside other social distancing measures, such as cancelations of religious events, partial curfew, banning of crowdedness activities, mass screening, and lockdown of shopping malls to minimize the risk of further virus transmission in the community.^14,15^

Concerning the preparedness of the health care system in KSA for the expected surge in health service demands during the pandemic, the Saudi facilities were prepared to receive large numbers of suspected and confirmed cases of COVID-19. The authority has designated reference hospitals, which reported their estimated isolation capacity and ICU beds, with their occupancy rates. Also, daily monitoring of the isolation bed occupancy rates was carried out to make all the necessary decisions to increase the isolation beds or transfer the uninfected patients to other hospitals.^16,17^ The ministry of health **(**MOH) has supplied an additional 3728 general hospital beds to field hospitals created to face the COVID-19 outbreak. It also created several field hospitals across all KSA regions and collaborated with various private sector hospitals. The number of extracorporeal membrane oxygenation machines in the ICU setting within the KSA is 24 machines. Aletreby et al. applied a Susceptible–Exposed–Infected–Recovered (SEIR) model compartments predictive model to forecast the maximum number of deaths, ICU admission, inpatient hospitalization, positive cases, and the time of their incidence caused by COVID-19.^11^ They showed that the model predicted a peak occurrence of the epidemic around the end of July 2020. The peak death was expected at 99 749 individuals. The projected peak of ICU admission was estimated as 70 246 patients and peak inpatient hospitalization of 11 997 936 persons. All these forecasted numbers were out of a sum of expected 14 049 105 COVID-19-positive confirmed cases.^11^ These predicted numbers and the actual numbers would burden and strain the health system surge capacity.

Therefore, providing an accurate estimate and assessment of inpatient general and ICU hospital bed surge capacity is vital for health care providers and governments to deal with the COVID-19 epidemic effectively. This is particularly crucial in terms of future planning, mobilization, and resource allocation for the response.^2,3,7^ We aimed to estimate and assess the health care system capacity in KSA to deal with the current COVID-19 epidemic. This study focused on the hard component of health system surge capacity, particularly the general and ICU hospital bed surge capacity. The current assessment will provide recommendations to the MOH that might be useful to adopt appropriate strategies in response to the COVID-19 pandemic.

## Materials and Methods

### Data

The present study used the data of general hospital beds, ICU beds, and ventilators from the National Health Emergency Operation Center WebEOC software controlled by the MOH. The software includes data regarding public and private sector medical care hospitals and centers across all KSA regions.

The data were collected and analyzed using an Excel spreadsheet template to assess the hospital beds and ICU bed surge capacity. This study only counted hospital beds accompanied by oxygen supply since caring for COVID-19 patients essentially requires oxygen treatment. On the other hand, this study only considered ICU beds accompanied by mechanical ventilators because mechanical ventilators are a fundamental and essential need to care for critical and severe COVID-19 cases. However, the available data did not provide information regarding the functionality of these and whether they were staffed and equipped or not.

### Methodology

To estimate the general hospital and ICU bed surge capacity, the authors computed 4 different hospital surge capacity measures for COVID-19, which were used from a previously published article in Kenya.^7^ The first measure pertained to the number of general hospital beds, which was defined as the percentage of available general hospital beds accompanied by oxygen suppliers that would be used to care for COVID-19 patients in KSA:(1)
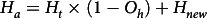



Where 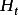 is the number of all general hospital beds, 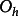 is the mean general hospital beds occupancy rate, and 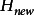 is the number of new hospital beds that were made available to face the COVID-19 pandemic in terms of field hospitals.

We followed the same procedure to estimate the ICU bed surge capacity, which is defined as the percentage of ICU beds with mechanical ventilators that would be used to care for COVID-19 patients in KSA:(2)
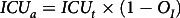



Where 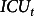 is the number of all ICU hospital beds and 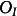 is the mean ICU bed occupancy rate.

The second measure estimated the number of persons that would be required for hospital admission (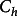) and critical care (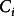):(3)
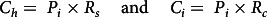



Where 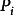 is the number of persons who are likely to get COVID-19 infection symptoms in the KSA population. The authors assume that about 2% of the Saudi population will be tested positive for COVID-19. 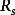 is an age-specific severe disease risk and 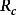 is an age-specific critical disease risk. These rates were obtained from a previously published study by the US Centers for Disease Control and Prevention (CDC) reporting team. Accordingly, the CDC reporting team had estimated that 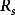 and 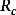 ranged from 20.7 to 31.4% and 4.9 to 11.5%, respectively.^18^ We used the upper values in the present study.

The third measure determined the number of general hospital bed days (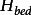) and the number of ICU bed days (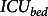):(4)
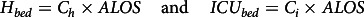



Where 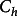 and 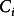 are defined in Equation 3, and 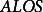 is the average length of stay in the hospital, estimated to be 12 days.^10^

The fourth measure determined the percentage of available general hospital beds (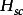), as well as the percentage of available ICU beds (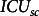) that would be used under different types of transmission scenarios (eg, 6, 12, and 18 months):(5)
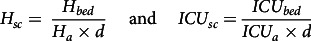



Where 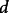 is the scenario duration in months.
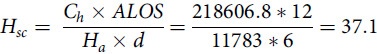


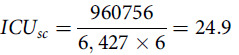



Finally, the authors determined the hospital bed tipping (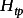) and ICU bed tipping (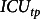) points, as follows:(6)
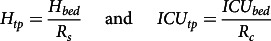



The tipping points were defined as the critical thresholds for each phase. Accordingly, 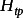 measures the maximum number of confirmed COVID-19 symptomatic infections that can be hospitalized by the existing number of general hospital beds in KSA. Similarly, 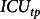 measures the maximum number of confirmed COVID-19 symptomatic infections that can be hospitalized by the existing number of ICU beds in KSA.

## Results

### General Hospital Bed and ICU Bed Surge Capacity

The number of current general hospital beds with oxygen supply is 58 899 beds (169 beds per 100 000 population). Out of them, 55 171 beds were already functioning across all hospitals (public and private), and 3728 beds accompanied by oxygen supply were embedded in field hospitals in response to COVID-19. Meanwhile, the total number of ICU beds accompanied by mechanical ventilators is 9181 (26.4 beds per 100 000 population) across all KSA hospitals. The average hospitalization rate for symptomatic COVID-19 patients in KSA is 85.4%,^11^ whereas the average baseline occupancy rate for ICU beds in KSA is 30%.^19^

The average number of hospital beds that would be used to care for COVID-19 patients is estimated to be 11 783 hospital beds (see Equation 1). The average number of ICU beds that would be used to care for COVID-19 patients is estimated to be 6427 ICU beds (see Equation 2).

**Figure 2.**
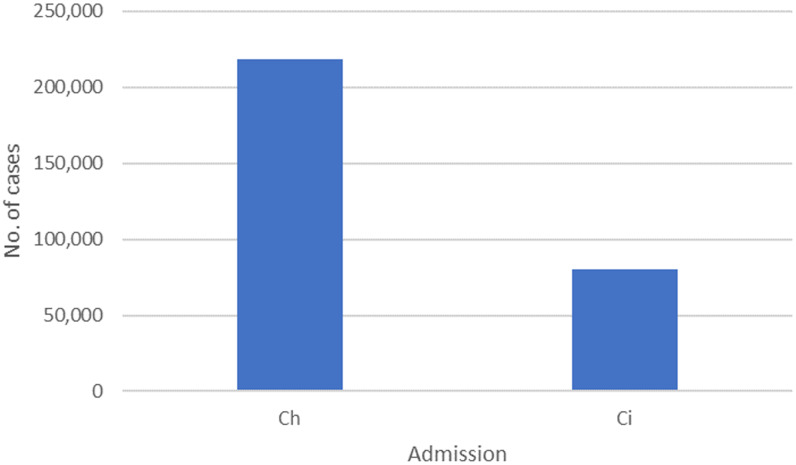
Number of persons that would be required for hospital admission (Ch) and critical care (Ci) assuming that 2% of the population would have COVID-19 symptoms.

The total population size in KSA for the year 2020 is 34 810 000.^11^ Assuming that about 2% of Saudis will be tested positive for COVID-19, the total number of persons who are likely to get COVID-19 infection symptoms among the KSA population is estimated to be 696 200 individuals. Therefore, the number of persons that would be required for hospital admission (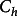) is 218 607 patients and critical care (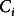) is 80 063 patients, as shown in Figure 2. Assuming that the average length of stay is 12 days,^20^ then the number of general hospital bed days (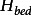) is estimated to be 2 623 284 general hospital beds and the number of ICU bed days (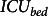) is estimated to be 960 756 ICU beds.

**Figure 3.**
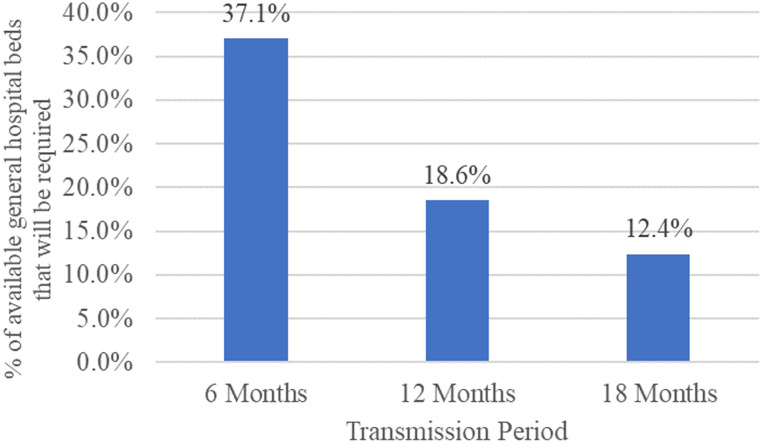
The percentage of available hospital beds would be required if 2% of the population are infected and develop symptoms.

**Figure 4.**
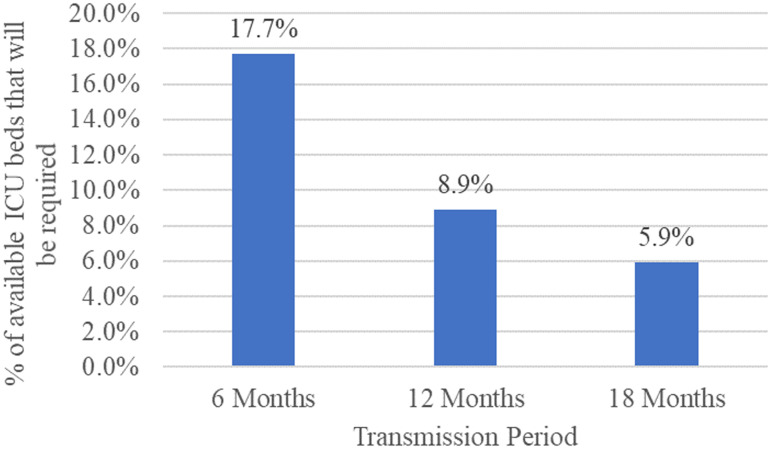
The percentage of available ICU beds would be required if 2% of the population are infected and develop symptoms.

The results also indicate that the percentages of available general hospital beds (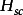) that would be used to care for potential COVID-19 cases under the 3 transmission scenarios – 6, 12, and 18 months – are 37.1%, 18.6%, and 12.4%, respectively, as shown in Figure 3. In other words, these percentages indicate that COVID-19 patients would occupy 37.1%, 18.6%, and 12.4% of the available number of general hospital beds in KSA if the outbreak persists up to 6, 12, and 18 months, respectively. Thus, hospitals in KSA need to be supplied with an additional 4372 (12.6 beds per 100 000 population) hospital beds to care for COVID-19 positive cases if the pandemic continues over a 6-month period. In the meantime, if the pandemic persists for 12 to 18 months, hospitals in KSA would require an extra 2192 (6.3 beds per 100 000 population) or 1461 (4.2 beds per 100 000 population) hospital beds, respectively. The corresponding percentages of available ICU beds (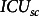) that would be used are 24.9%, 12.4%, and 8.3% of available ICU beds if the epidemic continues for 6, 12, and 18 months, respectively, as shown in Figure 4. Therefore, hospitals in KSA need to be supplied with an additional 1600 (4.6 beds per 100 000 population) ICU beds if the pandemic persists for a 6-month period. Meanwhile, if the pandemic persists for 12 to 18 months, hospitals in KSA would require an extra 797 (2.3 beds per 100 000 population) or 540 (1.6 beds per 100 000 population) ICU beds, respectively. The required additional general and ICU hospital beds were obtained based on Equation 6.

The results also reveal that the tipping points of general hospital beds vary from 4.8 million occurrences of infection symptoms when the epidemic continues over 6 months to 14.9 million occurrences when it persists over 18 months. On the other hand, ICU bed tipping points vary from 54 900 occurrences of infection symptoms when the epidemic continues for 6 months to 162 840 cases of infection symptoms when it continues to spread out over 18 months. These numbers exhibit the maximum number of cases that would be clinically treated using the general hospital and ICU bed surge capacity under different transmission situations (Table 1).

**Table 1.** General hospital bed and ICU bed tipping points

Transmission Curve Scenario	Maximum number of symptomatic cases that can be accommodated by existing hospital bed capacity	Maximum number of symptomatic cases that can be accommodated by existing ICU bed capacity
6 months	4,831,094	54,900
12 months	9,680,015	107,950
18 months	14,893,282	162,840

## Discussion

The global outbreak of COVID-19 has strained the health care systems across the world. The sudden fall down of surge capacity in some well-resourced countries – China, Italy, Iran, and Spain – alarmed most countries worldwide to assess and increase the health system surge capacity to respond to the COVID-19 pandemic accurately. Besides, countries should take critical steps to reduce infection rates, such as movement restrictions, quarantine, and social distances. This study aimed to evaluate the health system surge capacity in KSA, specifically in terms of the general hospital bed and ICU bed surge capacity to ingest and absorb the sudden increase in the number of COVID-19 cases. Our main finding is that KSA has no surge capacity for intensive care if the transmission curves would be flattened to 6, 12, or 18 months. There is some evidence of adequate surge capacity of general hospital beds if the transmission curve would be flattened to 12 or 18 months; however, this is not the case for a 6 months’ transmission scenario. Nonetheless, it should be noted that measuring health system surge capacity using only hospital beds data is insufficient. Measuring hospital beds capacity provides a partial picture of surge capacity, is an inadequate proxy of hospital capacity, and gives only a partial view of health system capacity. An adequate measure of health system surge capacity involves different elements, including human resources, equipment, infrastructure, and management.^5,7,21,22^ For example, the adequate capacity of general hospital and ICU beds should be supplemented by well-trained health workers and medical supplies for case management.^5,7^ Therefore, the current estimate of health system capacity is underestimated since it does not consider other components of health system surge capacity, which is the main limitation of the present study. However, this might provide a brief overview of the needs of the health system for hospital and ICU beds to face the COVID-19 pandemic.

The COVID-19 pandemic is a multidimensional problem that burdens the health system. It requires various crucial actions, including the capacity to test, map and isolate contacts, and serve health care employees with personal protective equipment. In KSA, the MOH reported the presence of 93.1 physicians and 206.9 nurses per 100 hospital beds in 2019. Moreover, there were 33.1 physicians, 58.2 nurses, and 22.5 hospital beds per 10 000 population.^19^ This indicates the capacity gaps between human resources and essential medical facilities in KSA, despite the proposed plan to have 2.0 to 2.7 hospital beds per 1000 population by 2030.^23^ A simple illustration of these gaps is, considering we have adequate hospital bed surge capacity to absorb extra COVID-19 cases, the lack of available health workers cannot overcome this issue. KSA has experienced similar challenges, such as Middle East respiratory syndrome (MERS) emerging in 2012.^24,25^

Furthermore, the 3 transmission scenarios in the current study assumed a constant infection rate; however, the true infection rates may be higher or lower.^7^ The estimates of maximum symptomatic infection over the 3 scenarios, especially the 18 months’ transmission scenario, are to some extent confirmed with other previously published predictive modeling studies applied to the KSA case. For instance, Aletreby et al. showed the expected peak occurrence of the epidemic to be around the end of July 2020. The peak death was expected to be 99 749 individuals, the projected peak of ICU admission was estimated as 70 246 patients, and the peak inpatient hospitalization was estimated to be 11 997 936 persons. These forecasted figures were out of a sum of expected 14 049 104.83 COVID-19-confirmed cases, and the pandemic is expected to continue for 18 to 24 months.^11^ The latter figure is almost consistent with our findings under the 18 months’ transmission scenario.

Despite the limitations of the current analysis, the results indicate that the health system in KSA would meet significant challenges if the COVID-19 pandemic continues to spread out. The findings of the current study also shed light on the importance of analyzing all surge capacity elements, if available, to decelerate the spread infection rate, which might reduce the overwhelming demand on the health care system.^7,26^ The present study results also necessitate interventions to enhance and expand the health care system surge capacity via adjusting health system measures and making the required capacities available. The MOH can use the experience of other countries to adjust and expand hospital bed surge capacity.^26^ This includes postponing admission to unnecessary patients, discharging ward patients after assessing them critically, making certain hospitals or closed guards, and creating field hospitals as centers for treatment and isolation of COVID-19 patients and contact trace.^7,26^ In fact, the MOH has taken some of these actions, but the pandemic still infects many people. For example, the MOH created several field hospitals across all KSA regions and collaborated with various private sector hospitals.

Besides the adjustment of surge capacity measures, immediate action by the MOH is of paramount importance to make more general hospital beds available to care for COVID-19 patients and activate the functionality of these beds with their accompanied oxygen suppliers. Such measures are more beneficial and realistic before focusing on providing new additional ICU beds accompanied by ventilators; new ICU beds would require training of the health workers, which is not feasible during the pandemic.^7,10^ The Saudi MOH supplied the field hospitals with only general beds (eg, 3728 beds), rather than including some ICU beds. Therefore, this improvement in the health system is vital to enhance the functionality of available ICU beds and provide additional ICU beds with ventilators to overcome the gaps in ICU bed surge capacity even though all current ICU beds are accompanied by ventilators.^7,27^

The analysis of health system surge capacity has been extensively debated in the literature. Results from most countries, including the United States, Australia, India, Italy, and Kenya, indicated a low adequate to inadequate general hospital and ICU beds capacity and insufficient staffing in the face of the COVID-19 pandemic.^7,10,21,26,28–32^ For example, the Kenyan health system surge capacity is somewhat adequate concerning the general hospital beds. At the same time, it suffers from inadequate ICU and ventilator capacities, which is almost similar to the KSA experience.^7^ Moreover, Carenzo et al. indicated that Italy has gradually experienced a critical care crisis, defined as a shortage of ICU beds and trained personnel.^31^ However, Cammarota et al. developed a strategy applied to the Novara hospital in Italy and indicated an increase in ICU bed surge capacity by 107%.^32^

Finally, this study’s results provide the government and the MOH with some necessary information on the magnitude of extra hospital bed surge capacity required for potential new gradual growth after some of the large-scale interventions at a later stage. As our investigations provide a partial view about health care surge capacity, further research is needed to evaluate all elements of health system surge capacity, which could provide a more comprehensive view of the health care system in KSA to respond appropriately to the COVID-19 pandemic.^33^

Despite the importance of ensuring adequate hospital beds’ capacity in the face of the COVID-19 pandemic, the role of preventive and mitigation strategies should not be overlooked. Preventive measures represent the cornerstone in managing the pandemic, including quarantine and lockdown measures, facial masks, hand hygiene, and self-isolation in suspected cases.^34,35^ The Saudi authority has implemented a wide range of preventive strategies to minimize the risk of COVID-19 transmission.^14,15^ Besides, the current evidence indicates that selected patients with severe COVID-19 can respond well to non-invasive oxygen delivery measures without the need for mechanical ventilation.^36^ Thus, treating physicians should be aware of the criteria of patients who do not need invasive mechanical ventilation, which can significantly reduce the need for ICU beds with mechanical ventilation.

## Conclusion

This study aimed to assess the general hospital and ICU bed surge capacity in KSA. Our findings highlight the urgent need for additional hospital and ICU beds in the face of critical COVID-19 cases in KSA. The study recommends further assessment measures to the health system surge capacity to keep the Saudi health system prepared during the COVID-19 pandemic.
